# Circular RNA PLCE1 promotes epithelial mesenchymal transformation, glycolysis in colorectal cancer and M2 polarization of tumor-associated macrophages

**DOI:** 10.1080/21655979.2021.2003929

**Published:** 2022-03-29

**Authors:** Bo Yi, KeJu Dai, ZhiQiang Yan, ZhaoHui Yin

**Affiliations:** a Gastrointestinal Surgery, University of Eletronic Science and Technology of China, Chengdu City, SiChuan Province, China; b Ultrasonic Medical Center, University of Eletronic Science and Technology of China, ChengDu City, SiChuan Province, China; c Department of Anus and Intestine Surgery, The Affiliated Hospital of GuiZhou Medical University, GuiYang City, GuiZhou Province, China; d Department of Anus and Intestine Surgery, The Affiliated Maotai Hospital of ZunYi Medical University, ZunYi City, GuiZhou Province, China

**Keywords:** Colorectal cancer, circular RNA PLCE1, microRNA-485-5p, epithelial mesenchymal transformation, glycolysis, tumor-associated macrophage polarization

## Abstract

Plentiful studies have clarified that circular RNAs (circRNAs) are crucial in colorectal cancer (CRC)’s occurrence and development, but its function has not been fully elucidated. The purpose of this study was to investigate the biological functions of circPLCE1 on epithelial mesenchymal transformation (EMT) and glycolysis in CRC, and tumor-associated macrophage (TAM) polarization. The results affirmed augment of circPLCE1 and γ-Actin Gene (ACTG1) but decline of miR-485-5p in CRC. Knockdown circPCLE1 refrained CRC proliferation, glucose consumption, lactic acid and pyruvate production, M2 macrophage markers (IL-10, MRC1), N-cadherin, Snail, reduced the proportion of CD206+ and CD168+ macrophages, but expedited M1 macrophage markers (TNF-α, IL-6) and E-cadherin, while descending miR-485-5p expedited EMT, glycolysis in CRC and TAM M2 polarization . Additionally, it was affirmed that the repression or motivation of depressive or elevated circPCLE1 on EMT, glycolysis in CRC and TAM M2 polarization were reversed via facilitated ACTG1 and miR-485-5p, separately. Mechanism studies have clarified that circPCLE1 as a competitive endogenous RNA adsorbed miR-485-5p to mediate ACTG1. It was assured that refrained circPCLE1 constrained CRC tumor growth, EMT and TAM M2 polarization. In brief, circPCLE1 expedites EMT, glycolysis in CRC and TAM M2 polarization via modulating the miR-485-5p/ACTG1 axis, and is supposed to be a latent molecular target for CRC therapy later.

## 1 Introduction

Colorectal cancer (CRC) is a common malignant tumor of the digestive system and remains a huge public health challenge worldwide [1]. Among malignancies, CRC takes on a surprising incidence and mortality rate, ranking the third and second separately [2]. Nowadays, CRC therapy mainly contains radiotherapy, surgery, chemotherapy, and other adjuvant therapies, while the impact is not ideal with the unpleasing prognosis [3]. Hence, it is vital to have a deeper understanding of the pathogenesis of CRC and to seek brand-new therapeutic targets.

CRC cell behaviors are concentrated on in most former studies, consisting of proliferation, migration and invasion CRC [4,5]. Plentiful studies have clarified that changes in tumor microenvironment is crucial in the malignant metastasis and proliferation of CRC [6,7]. Tumor-associated macrophages (TAM) in tumor microenvironment are more likely to polarize into M2 kind and expedite metastasis, tumor proliferation, migration, and angiogenesis [8,9]. In the meantime, epithelial–mesenchymal transformation (EMT) is an crucial reason of tumor invasion and metastasis [10,11]. During EMT, epithelial phenotype is lost in the basal epithelial cells to gain mesenchymal phenotype with the cell characteristics of descending E-cadherin, strengthened N-cadherin and Snail, and destruction of extracellular matrix, which is momentous in the stage of tumor metastasis [12,13]. A great many energy is needed in tumor progression and metastasis, and glycolysis is the main source of energy for tumor. Nevertheless, the mechanisms underlying TAM polarization, EMT and glycolysis in CRC have not been entirely expounded [14,15].

Circular RNAs (circRNAs), a kind of non-coding RNA, are elevated and stable in tissues and cells [16]. Recently, plenty of studies have elucidated the role of circRNA in the malignant metastasis and proliferation of CRC. For instance, circ3823 is discrepantly expressed in CRC and expedites its angiogenesis, growth, and metastasis [17]. Meanwhile, circRHOBTB3 restrains CRC metastasis via mediating the mRNA stability of the HUR-mediated Polypyrimidine tract-binding protein 1 [18]. CircMFN2 expedites CRC radiation resistance, proliferation and metastasis via controlling the microRNA (miR)-574-3p/the type 1 insulin-like growth factor receptor pathway [19]. Recently, Chen Z *et al.* reported that a brand-new circRNA PLCE1 expedites CRC proliferation and migration [20]. Nevertheless, the character of circPLCE1 on TAM polarization, EMT and glycolysis in CRC tumor microenvironment is still ambiguous.

In this study, it was examined the expression of circPLCE1 in CRC tissues and cells and confirmed its clinical significance. In addition, it was explored the effects of circPLCE1 on TAM M2 polarization, EMT and glycolysis of CRC cells through functional gain and loss experiments. Finally, bioinformatics and functional rescue experiments were applied to identify the underlying molecular mechanisms in which circPLCE1 influenced CRC progression via modulating miR-485-5p/γ-Actin Gene (ACTG1) axis.

## 2 Materials and methods

### 2.1 Clinical samples

From April 2017 to January 2020, CRC patients admitted to Maotai Hospital affiliated to Zunyi Medical University (n = 38) were included. No acceptance of preoperative chemotherapy or radiotherapy was in the patients. During tumor resection, collection of fresh tumor and paired para-cancer tissues was manifested. Confirmation of all resected tissues was via pathology. Storage of the tissue was in liquid nitrogen at −80°C for subsequent research. The research methods lived up to the criteria set out in *the Declaration of Helsinki*. Approval of this study was via the Ethics Committee of Maotai Hospital affiliated to Zunyi Medical University. Informed consent has been gained from all patients in this study.

### 2.2 Cell culture

Culture of human normal colonic epithelial cell-line NCM460, human monocyte THP-1 cells and CRC cell line SW480 (Cell resource center, SIBS, CAS, Shanghai, China) was separately in Roswell Park Memorial Institute-1640 medium (Gibco, Invitrogen, Carlsbad, CA, USA), Dulbecco’s Modified Eagle’s medium (Gibco) with routine supplementation of 10% fetal bovine serum (FBS) (Hyclone, Logan, UT, USA). Differentiation of THP-1 monocytes was with 150 nm phorbol 12‐myristate 13‐acetate (PMA) (Sigma-Aldrich, St Louis, MO, USA) into macrophages.

### 2.3 Reverse transcription quantitative polymerase chain reaction (RT-qPCR)

Conduction of RT-qPCR was as set forth [21]. In short, extraction of total RNA of the sample was with Trizol reagent (Takara, Otsu, Japan). Conduction of reverse transcriptase reaction was with HiScript® III 1^st^ Strand cDNA Synthesis Kit (Vazyme, Nanjing, China). Employment of SYBR green reagent (Vazyme, Nanjing, China) and RT-qPCR assay system (Analytic, Jena, Germany) was in all RT-qPCR tests. Quantitative measurements were gained via 2^−ΔΔCT^ and normalized to glyceraldehyde-3-phosphate dehydrogenase (GAPDH) or U6 levels. Primer sequences are clarified in Table 1.

**Table 1.** Primer sequences of RT-PCR

	Primer sequences (5’ – 3’)
GAPDH	Forward: 5’-GGGAGCCAAAAGGGTCAT-3’
	Reverse: 5’-GAGTCCTTCCACGATACCAA-3’
U6	Forward: 5’-CTCGCTTCGGCAGCACA-3’
	Reverse: 5’-AACGCTTCACGAATTTGCGT-3’
circPLCE1	Forward: 5’-TACAGGACAGTCAGTGGTGGTA-3’
	Reverse: 5’-ACATCTAGCTTAGGAATGTGGC-3’
MiR-485-5p	Forward: 5’- AGAGGCTGGCCGTGAT −3’
	Reverse: 5’- GAACATGTCTGCGTATCTC −3’
ACTG1	Forward: 5’- ATGGAAGGAAACACGGCTC −3’
	Reverse: 5’- CACTCTGTTCTTCCGCCG −3’
TNF-α	Forward: 5’-CCCTCACACTCAGATCATCTTCT-3’
	Reverse: 5’-GCTACGACGTGGGCTA CAG-3’
IL-6	Forward: 5’-TCCATCCAGTTGCCTTCTTGA-3’
	Reverse: 5’-AAGCCTCCGACTTGTGAAGTGC-3’
IL-10	Forward: 5’- GTCATCGATTTCTTCCCTGTG −3’
	Reverse: 5’- ACTCATGGCTTTTGTAGATGCCT −3’
MRC1	Forward: 5’- GGCGGTGACCTCACAAGTAT −3’
	Reverse: 5’- ACGAAGCCATTTGGTAAACG −3’

### 2.4 Cell transfection

For gaining SW480 cells with stable elevated circPLCE1/miR-485-5p/Mutations in the γ-Actin Gene (ACTG1), application of Lipofectamine 3000 (Invitrogen) was to transfect pcDNA3.1, pcDNA3.1-circPLCE1, mimic NC, miR-485-5p mimic and pcDNA 3.1-ACTG1 into SW480 cells with offered manuals. For obtaining SW480 cells with stable silenced circPLCE1/miR-485-5p, Lipofectamine 3000 (Invitrogen) was given application to transfect si-NC, si-circPLCE1, inhibitor NC and miR-485-5p inhibitor into SW480 cells. Then collection of the cells was for subsequent experiments. Detection of the transfection efficiency was via RT-qPCR. Design and supply of the plasmids or oligonucleotides was via GenePharma (Shanghai, China).

### 2.5 Acquisition of conditioned medium (CM)

For gaining CM for SW480 cells, wash of SW480 cells with 80% confluence was in FBS-free medium and maintain in fresh FBS-free medium. Treatment of THP-1-differentiated macrophages was with CM of SW480 cells.

### 2.6 Colony formation

Detection of cell proliferation was via Colony Formation [22]. Seeding of SW480 cells was in 6-well plates at a density of 5 × 10^3^ cells per well with changed medium every 3 d. After removing the medium and the unbound cells, fixation of adherent cells was with 4% paraformaldehyde and stain was with 0.01% crystal violet. Calculation of colonies of over 50 cells was manifested. N = 3.

### 2.7 Flow cytometry

Detection of cell apoptosis was via Annexin V-fluorescein isothiocyanate kit (Thermo Fisher Scientific, Inc.) [23]. Collection of SW480 cells, resuspension in cold phosphate buffered saline (PBS), centrifugation, incubation with 5 μL Annexinv-Alexa Fluor 647 and then with 5 μL propidium iodide before detection were implemented. Finally, analysis of the apoptosis rate was via FACSCalibur flow cytometer (BD Biosciences, Franklin Lakes, N).

For examination of CD206+ and CD163+ cell levels, there were detachment of macrophages with trypsin, suspension in PBS, and treatment with human anti-CD206 (18,704-1-AP, Proteintech), monoclonal antibody CD163 (ab182422, Abcam) coupled with 1 μg/mL fluorescein isothiocyanate. Analysis of CD206+ and CD163+ cell levels was via flow-cytometry FACSCalibur flow cytometer (BD Biosciences, Franklin Lakes, N).

### 2.8 Glycolysis analysis

Determination of pyruvate and lactate production and glucose consumption in SW480 cells was via the pyruvate, lactic acid, and glucose detection kits (ab65342, ab65331, ab136955, Abcam) obeying the manufacturer’s agreement.

### 2.9 Western blot

Conduction of Radio-Immunoprecipitation assay (RIPA) lysis buffer (Beyotime, Shanghai, China) and Bradford Protein Assay Kit (Beyotime) was for protein extraction and concentration measurement. Isolation via 10% sulfate polyacrylamide gel electrophoresis of the protein sample (15 μg) and electroblot onto Polyvinylidene fluoride membrane (Millipore, Bedford, MA, USA) were implemented. Then incubation of the membrane was with 5% skim milk and test was with primary antibodies, consisting of ACTG1 (4968), E-cadherin (3195), N-cadherin (13,116), Snail (3879), GAPDH (2118) (all Cell Signaling Technology). Detection of the membrane was with horseradish peroxidase-coupled secondary antibody (Beyotime), followed by visualization with electrogenerated chemiluminescence reagent (Millipore). Application of GAPDH was as a loading control gene.

### 2.10 The luciferase activity assay

Dual-luciferase reporting assay was performed as described previously [24]. Subclone of wild/mutant type reporter vectors (ACTG1/circPLCE1-WT/MUT) (Synthgene Biotech, Nanjing, China) was into pmirGLO vector (Promega, Madison, WI, USA). Co-transfection of the vectors was with miR-485-5p mimic or its NC into SW480 cells via lipofectamine 3000 (Invitrogen). Measurement of the relative luciferase activity of the cells was via a dual Glo luciferase assay system (Promega, Shanghai, China) with introduction of the manufacturer. Normalization of the luciferase activity was via Renilla signals.

### 2.11 RNA immunoprecipitation (RIP) assay

With the manufacturer’s instructions, application of the EZ-Magna RIP kit (Millipore) was for RIP [25]. Lysis of SW480 cells was with RIPA lysis buffer consisting of a mixture of protease and phosphatase inhibitors (Sigma-Aldrich Chemical Company, St Louis MO, USA). Preincubation of magnetic beads (Invitrogen) was with anti-AGO2 (ab32381, Abcam) or anti-Immunoglobulin G (ab6721, Abcam). Immunoprecipitation of the magnetic beads was with the lysate. Purification of RNA was from RNA-protein complex and analysis was via RT-qPCR.

### 2.12 Xenograft in nude mice

Gain of 14 male 6-week-old athymic BALB/c nude mice was from SLAC Laboratory Animal Co. Ltd (Shanghai, China). Carrying out animal experiments was on the grounds of the guidelines of the Animal Care and Use Committee of Maotai Hospital affiliated to Zunyi Medical University. Subcutaneous injection of SW480 cells (2 × 10^6^ cells) of knockdown circPLCE1 was into the flank of nude mice. Weekly recording of tumor growth was with a vernier caliper. Calculation of tumor volume was as (a × b)^2^ × 0.5 (a, long axis; b, short axis). On the 28^th^ d after injection, euthanasia of the mice, resection of the tumor, photographing and weighing were implemented. After fixation of the tumor tissue with 4% paraformaldehyde and embedding in paraffin, Immunohistochemical analysis of tumor tissues was via antibodies against Ki-67, CD206 and CD163.

### 2.13 Immunohistochemistry

Performance of immunohistochemistry was as set forth [26]. Dewaxing of paraffin sections was in xylene and rehydration was via an alcohol gradient. After antigenic repair, seal of all sections was in an avidin/biotin blocking buffer (Vector Laboratories) and then in 3% Bovine serum albumin. Incubation of the sections was with primary antibody Ki-67 (ab15580, Abcam), CD206 (18,704-1-AP, Protein-tech) and CD163 (ab182422, Abcam). Protein staining was via Diaminobenzidine substrate kit (Maixin Biotech, Kit-9710). Counterstain of the samples was with hematoxylin. Gain of immunohistochemical images was via a forward microscope (Olympus BX51). Brown staining clarified immunoreactivity of the sample.

### 2.14 Data analysis

Expression of all data was as mean ± standard deviation (SD). Application of SPSS 18.0 (IBM, Armonk, NY, USA) and Graphpad Prism 9.0 was for data analysis and mapping, separately. Student’s t-test was for comparison of differences between the two groups, with Chi-square test for detection of differences in clinical features. *P* < 0.05 was manifested statistically significant. Biological replication of all experiments was at least three times.

## 3 Results

### 3.1 Elevated circPLCE1 is in CRC

**Figure 1.**
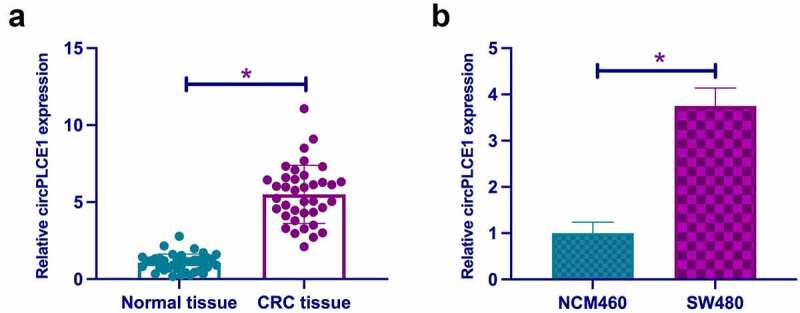
**Elevation of CircPLCE1 is in CRC** A. RT-qPCR to detect circPLCE1 in CRC and adjacent normal tissues; B. RT-qPCR to detect circPLCE1 in CRC cell line SW480 and human normal colonic epithelial cell line NCM460. Expression of the data was as mean ± SD (B, n = 3); *P < 0.05.

In this study, examination of circPLCE1 was in CRC, affirming the elevation in CRC tissues and cell line SW480 versus the adjacent normal tissues and human normal colonic epithelial cell-line NCM460 (Figure 1(a, b)), which was in agreement with a former report [20]. For examination of the relationship of circPLCE1 and clinicopathological features of CRC patients, assignation of the patients was into high and low circPLCE1 groups on the grounds of the median circPLCE1 expression. As clarified in Table 2, circPLCE1 was relevant to lymph node metastasis in CRC patients. All in all, circPLCE1 was up-regulated in CRC and connected with clinicopathology.

**Table 2.** CircPLCE1 is relevant to lymph node metastasis in CRC patients

Characteristic	Groups	n	CircPLCE1 expression		*P*
			High (n = 19)	Low (n = 19)	
Age (years)	< 60	11	4	7	0.2832
	60 or more	27	15	12	
Gender	Male	26	14	12	0.4852
	Female	12	5	7	
Tumor size (cm)	< 5	17	10	7	0.3277
	5 or more	21	9	12	
Lymph node metastasis	Yes	28	17	11	0.0271
	No	10	2	8	
TNM staging	I + II	8	5	3	0.4261
	III	30	14	16	

Note: TNM, Tumor–node–metastasis.

### 3.2 Knockdown circPLCE1 represses TAM M2 polarization, and CRC EMT and glycolysis

**Figure 2.**
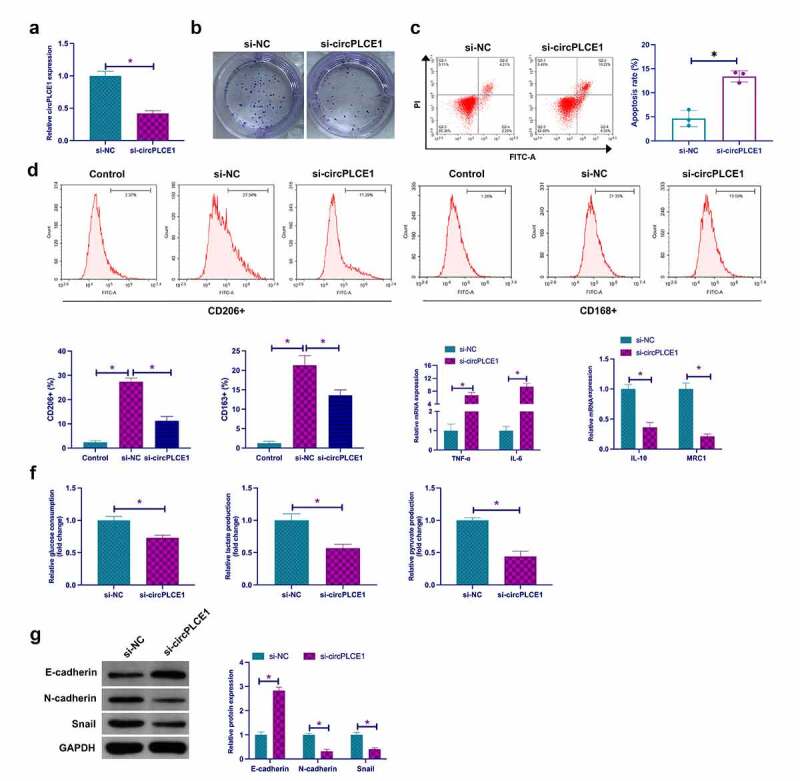
**Repressive circPLCE1 curbs TAM M2 polarization, and CRC EMT and glycolysis** a. RT-qPCR to detect circPLCE1 in SW480 cells transfected with si-circPLCE1; b. Colony formation detection of the impact of circPLCE1 suppression on SW480 cell proliferation; c. Flow cytometry to detect the impact of circPLCE1 suppression on apoptosis of SW480 cells; d. Flow cytometry to detect the impact of macrophages cultured with SW480 cells with silenced circPLCE1 and CM on the proportion of CD206+ and CD163+ cells; e. RT-qPCR to detect the impact of macrophages cultured with SW480 cells with silenced circPLCE1 and CM on M1 macrophage markers TNF-α and IL-6 and M2 macrophage markers IL-10 and MRC1; f. The impacts of suppressive circPLCE1 on glucose consumption, lactic acid and pyruvate production of SW480 cells; g. Western blot to detect the impacts of circPLCE1 suppression on E-cadherin, N-cadherin and Snail in SW480 cells. Expression of the data was as mean ± SD (n = 3); *P < 0.05.

Next, exploration of the biological function of circPLCE1 was in CRC. Transfection of siRNA targeting circPLCE1 was into SW480 cells to knock down circPLCE1, the success of which was testified (Figure 2(a)), clarifying that depressive circPLCE1 refrained SW480 cell proliferation but accelerated the apoptosis rate (Figure 2(b,c)). For exploration of the impact of circPLCE1 knockdown on macrophage polarization, culture of PMA-induced human monocyte THP-1 was with CM transfected with SW480 cells of si-circPLCE1. It was affirmed that circPLCE1 knockdown reduced the percentage of CD206+ and CD168+ cells in THP-1-derived macrophages (CD206 and CD168 are surface markers of M2 macrophages) (Figure 2(d)). In the meantime, detection of M1 macrophage markers TNF-α and IL-6, and M2 macrophage markers IL-10 and MRC1 affirmed that suppressive circPLCE1 elevated TNF-α and IL-6, but restrained IL-10 and MRC1 (Figure 2(e)). Glycolysis is a crucial approach for cancer cells to gain energy for growth and metastasis. Examination of the impact of circPLCE1 depression on CRC glycolysis was conducted. As clarified in figure 2(f), restrained circPLCE1 brought down glucose consumption, lactate and pyruvate production in SW480 cells. Then examination of the influence of circPLCE1 suppression on EMT was conducted. As clarified in Figure 2(g), depressive circPLCE1 motivated E-cadherin but restrained N-cadherin and Snail. Shortly, suppressive circPLCE1 effectively refrained TAM M2 polarization, and CRC EMT and glycolysis.

### 3.3 Descending miR-485-5p expedites TAM M2 polarization, and CRC EMT and glycolysis

**Figure 3.**
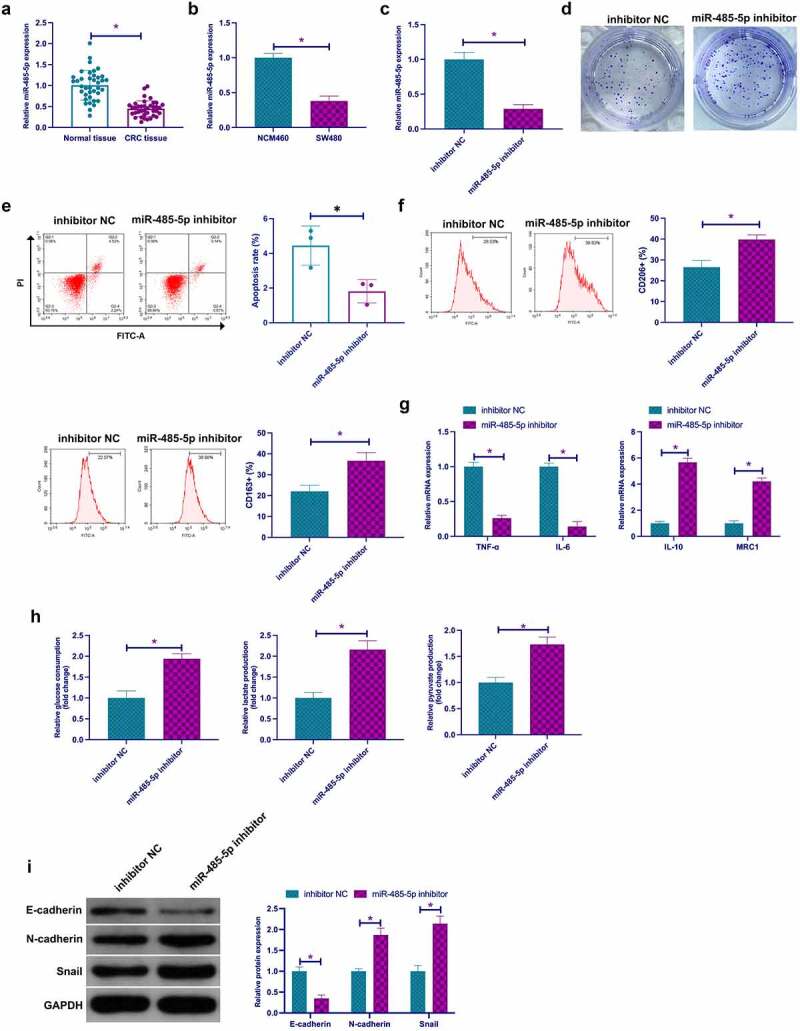
**Knockout miR-485-5p expedites TAM M2 polarization, and CRC EMT and glycolysis** a. RT-qPCR to detect miR-485-5p in CRC and adjacent normal tissues; b. RT-qPCR to detect miR-485-5p in CRC cell line SW480 and human normal colonic epithelial cell line NCM460; c. RT-qPCR to detect miR-485-5p in SW480 cells transfected with miR-485-5p inhibitor; d. Colony formation detection of the impact of miR-485-5p suppression on SW480 cell proliferation; e. Flow cytometry to detect the impact of miR-485-5p suppression on apoptosis of SW480 cells; f. Flow cytometry to detect the impact of macrophages cultured with SW480 cells with silenced miR-485-5p and CM on the proportion of CD206+ and CD163+ cells; g. RT-qPCR to detect the impact of macrophages cultured with SW480 cells with silenced miR-485-5p and CM on M1 macrophage markers TNF-α and IL-6 and M2 macrophage markers IL-10 and MRC1; h. The impacts of suppressive miR-485-5p on glucose consumption, lactic acid and pyruvate production of SW480 cells; I. Western blot to detect the impacts of miR-485-5p suppression on E-cadherin, N-cadherin and Snail in SW480 cells. Expression of the data was as mean ± SD (n = 3); *P < 0.05.

Former studies have affirmed that miR-485-5p serves as a tumor suppressor gene in CRC and blocks CRC proliferation and invasion [27,28]. Nevertheless, the role of miR-485-5p in CRC macrophage polarization, glycolysis, and EMT is uncertain. To this end, examination of the biological functions of miR-485-5p was in the fields of CRC. Firstly, examination of miR-485-5p in CRC clarified memorable decline in tissues and cells versus normal controls (Figure 3(a,b)). Subsequently, depressive miR-485-5p was manifested in SW480 cells via transfection with miR-485-5p inhibitor (Figure 3(c)). It was affirmed that depressive miR-485-5p facilitated colony formation and restrained apoptosis rate of SW480 cells (Figure 3(d,e)). Subsequently, culture of macrophages was with SW480 cells transfected with miR-485-5p inhibitor in CM, clarifying that repressive miR-485-5p motivated the percentage of CD206+ and CD168+ cells, IL-10, MRC1, glucose consumption, lactate and pyruvate production in SW480 cells, N-cadherin and Snail, but restrained TNF-α, IL-6, E-cadherin (figure 3(f-i)). In a word, miR-485-5p was reduced in CRC, and repressive miR-485-5p expedited TAM M2 polarization, and CRC EMT and glycolysis.

### 3.4 CircPLCE1 competitively adsorbs miR-485-5p

**Figure 4.**
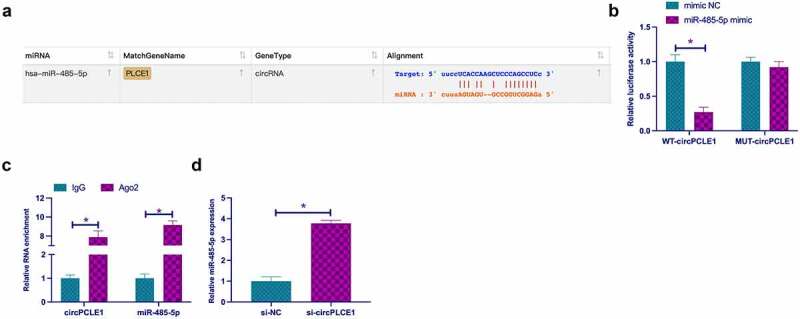
**CircPLCE1 competitively adsorbs miR-485-5p** a. The binding sites of circPLCE1 and miR-485-5p queried through the bioinformatics website; b. Dual luciferase reporting assay to examine the targeting of circPLCE1 and miR-485-5p; c. RIP test to check the targeting of circPLCE1 and miR-485-5p; d. RT-qPCR to detect miR-485-5p in SW480 cells after transfection with si-circPLCE1; Expression of the data was as mean ± SD (n = 3); *P < 0.05.

Next, it was explored the relationship of circPLCE1 with miR-485-5p. First of all, through bioinformatics website http://starbase.sysu.edu.cn/ was found that circPLCE1 and miR-485-5p had potential-binding sites (Figure 4(a)). Dual-luciferase reporting assay clarified that WT-circPLCE1 could reduce the luciferase activity in miR-485-5p mimic group, but MUT-circPLCE1 could not affect that in miR-485-5p mimic group (Figure 4(b)). In addition, the results were further verified via RIP assay. As manifested in Figure 4(c), enriched miR-485-5p and circPLCE1 were detected in the Ago2 group. In the meantime, it was found that knocking down circPLCE1 elevated the expression of miR-485-5p in SW480 cells (Figure 4(d)). These results suggested that miR-485-5p was modulated via circPLCE1 in CRC.

### 3.5 CircPLCE1 affects TAM M2 polarization, and CRC EMT and glycolysis via mediating miR-485-5p

**Figure 5.**
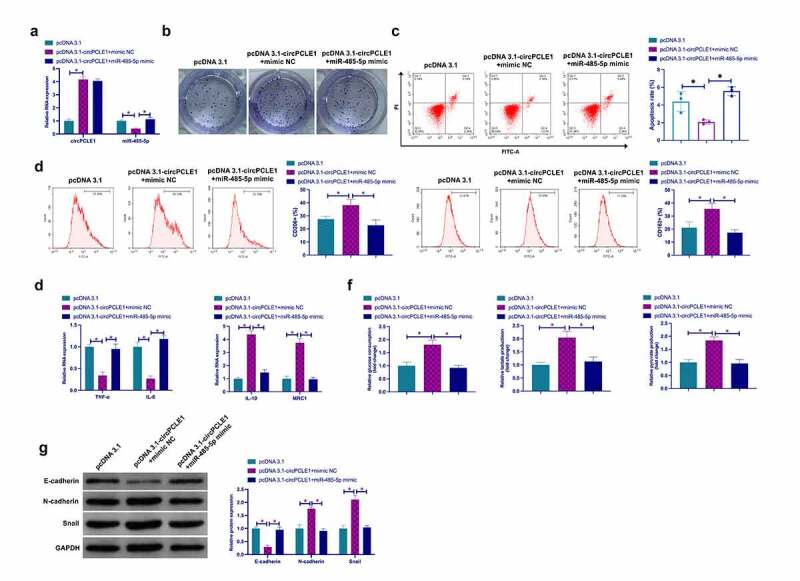
**CircPLCE1 influences TAM M2 polarization, and CRC EMT and glycolysis via controlling miR-485-5p** a. RT-qPCR to detect circPLCE1 and miR-485-5p after co-transfection of pcDNA 3.1-circPLCE1 and miR-485-5p mimic; b. Colony formation to detect the impacts of augmented circPLCE1 and miR-485-5p on SW480 cell proliferation; c. Flow cytometry to detect the impacts of augmented circPLCE1 and miR-485-5p on apoptosis of SW480 cells; d. Flow cytometry to detect the impact of macrophages cultured with SW480 cells with augmented circPLCE1 and miR-485-5p and CM on the proportion of CD206+ and CD163+ cells; e. RT-qPCR to detect the impact of macrophages cultured with SW480 cells with augmented circPLCE1 and miR-485-5p and CM on M1 macrophage markers TNF-α and IL-6, and M2 macrophage markers IL-10 and MRC1; f. The impacts of simultaneously elevated circPLCE1 and miR-485-5p on glucose consumption, lactic acid and pyruvate production in SW480 cells; g. Western blot to detect the impacts of augmented circPLCE1 and miR-485-5p on E-cadherin, N-cadherin and Snail in SW480 cells. Expression of the data was as mean ± SD (n = 3); *P < 0.05.

Next, whether miR-485-5p was involved in circPLCE1’s regulation of CRC was examined. For elevating circPLCE1 and miR-485-5p simultaneously, co-transfection of pcDNA 3.1 targeting circPLCE1 and miR-485-5p mimics was into SW480 cells with examination of the co-transfection efficiency (Figure 5(a)). It was affirmed that the motivated SW480 cell proliferation and repressive apoptosis via circPLCE1 elevation were reversed via the simultaneous strengthening miR-485-5p (Figure 5(b, c)). Subsequently, culture of THP-1 cells was with CM of co-transfected SW480, manifesting that augmented circPLCE1 accelerated the proportion of CD206+ and CD163+ cells, IL-10 and MRC1, glucose consumption, lactate and pyruvate production in SW480 cells, N-cadherin and Snail, but repressed TNF-α, IL-6 and E-cadherin that was reversed via motivated miR-485-5p (Figure 5(d-g)). In short, miR-485-5p was relevant to circPLCE1’s regulation of CRC.

### 3.6 ACTG1 is the target gene of miR-485-5p

**Figure 6.**
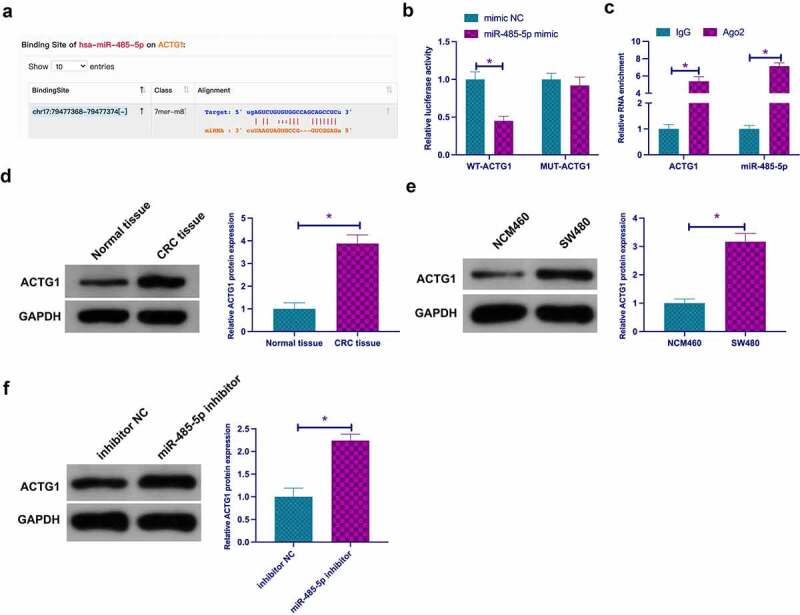
**ACTG1 is the target gene of miR-485-5p** a. The binding sites of miR-485-5p and ACTG1 predicted via bioinformatics websites; b. Dual luciferase reporting assay to check the targeting between ACTG1 and miR-485-5p; c. RIP assay to check the targeting between ACTG1 and miR-485-5p; D and E. ACTG1 in CRC tissues and cells detected via Western blot; f. Western blot to detect the impact of reduced miR-485-5p on ACTG1 in SW480 cells; Expression of the data was as mean ± SD (n = 3); *P < 0.05.

MiRNAs influence their biological functions via binding to downstream proteins. Next, exploration of the target genes of miR-485-5p was conducted. Via biological information website http://starbase.sysu.edu.cn/ was forecast that ACTG1 and miR-485-5p had latent-binding sites (Figure 6(a)). In order to further validate the conjecture, dual-luciferase reporting assay was carried out, and the results revealed that WT-ACTG1 reduced the luciferase activity in miR-485-5p mimic group, while MUT-ACTG1 had no effect on that in miR-485-5p mimic group (Figure 6(b)). In addition, RIP assay manifested that miR-485-5p and ACTG1 were apparently enriched in the Ago2 group (Figure 6(c)). Former studies have assured that ACTG1 is an oncogene in hepatocellular carcinoma and colorectal adenocarcinoma [29,30]. It was affirmed elevated ACTG1 in CRC tissues and cells versus normal controls (Figure 6(d)), which further supports the idea that ACTG1 is an oncogene in cancer. In the meantime, descending miR-485-5p strengthened ACTG1 (Figure 6(e)), manifesting that miR-485-5p modulated ACTG1 in CRC.

### 3.7 CircPLCE1 modulates TAM M2 polarization, and CRC EMT and glycolysis through miR-485-5p/ACTG1 axis

**Figure 7.**
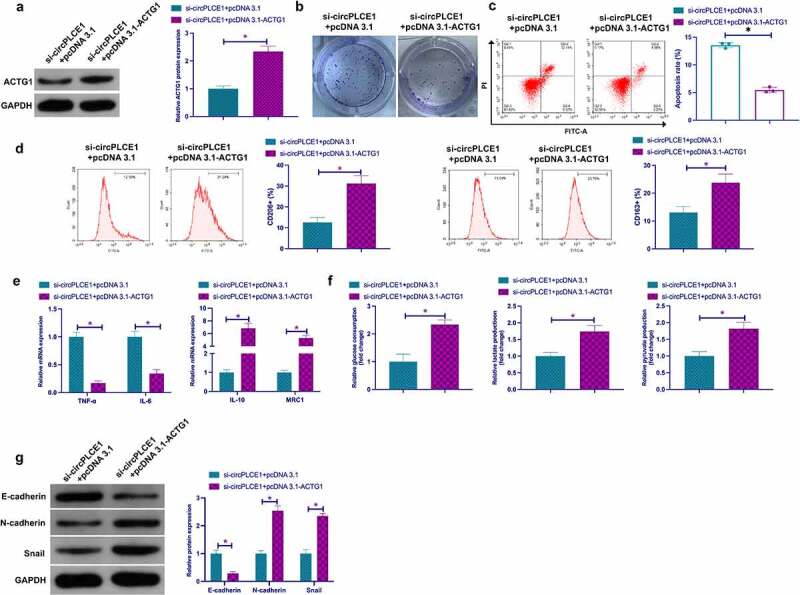
**CircPLCE1 modulates TAM M2 polarization, and CRC EMT and glycolysis through the miR-485-5p/ACTG1 axis** a. Western blot to detect the impact of co-transfected si-circPLCE1 and oe-ACTG1 on ACTG1 in SW480 cells; b. The impact of simultaneous circPLCE1 knockdown or ACTG1 elevation on SW480 cell proliferation detected via colony formation assay; c. Flow cytometry to detect the impacts of simultaneous circPLCE1 knockdown or ACTG1 elevation on apoptosis of SW480 cells; d. Flow cytometry to detect the impact of macrophages cultured with SW480 cells with simultaneous circPLCE1 knockdown or ACTG1 elevation and CM on the proportion of CD206+ and CD163+ cells; e. RT-qPCR to detect the impact of macrophages cultured with SW480 cells with simultaneous circPLCE1 knockdown or ACTG1 elevation and CM on M1 macrophage markers TNF-α and IL-6, and M2 macrophage markers IL-10 and MRC1; f. The impacts of simultaneous knockdown of circPLCE1 or elevation of ACTG1 on glucose consumption, lactic acid and pyruvate production in SW480 cells; g. Western blot analysis of the impacts of simultaneous circPLCE1 knockdown or ACTG1 elevation on E-cadherin, N-cadherin and Snail in SW480 cells; Expression of the data was as mean ± SD (n = 3); *P < 0.05.

Next, whether circPLCE1 regulated CRC through miR-485-5p/ACTG1 axis was examined. Co-transfection of si-circPLCE1 and pcDNA3.1-ACTG1 was into SW480 cells. It was affirmed that circPLCE1 depression reduced ACTG1, while coinstantaneous elevation of ACTG1 facilitated ACTG1 (Figure 7(a)), and motivated ACTG1 prevented circPLCE1 knockdown from descending proliferation and strengthened apoptosis (Figure 7(b, c)). Subsequently, culture of macrophages derived from THP-1 cells was with CM of the co-transfected SW480 cells, assuring that repressive circPLCE1 refrained the proportion of CD206+ and CD163+ cells, IL-10 and MRC1, glucose consumption, lactate and pyruvate production in SW480 cells, N-cadherin and Snail, but augmented TNF-α, IL-6 and E-cadherin, which was reversed via motivated ACTG1 (Figure 7(d-g)). In short, circPLCE1 modulated TAM M2 polarization, and CRC EMT and glycolysis through the miR-485-5p/ACTG1 axis.

### 3.8 *Depressive circPLCE1 represses tumor growth, TAM M2 polarization, and CRC EMT* in vivo

**Figure 8.**
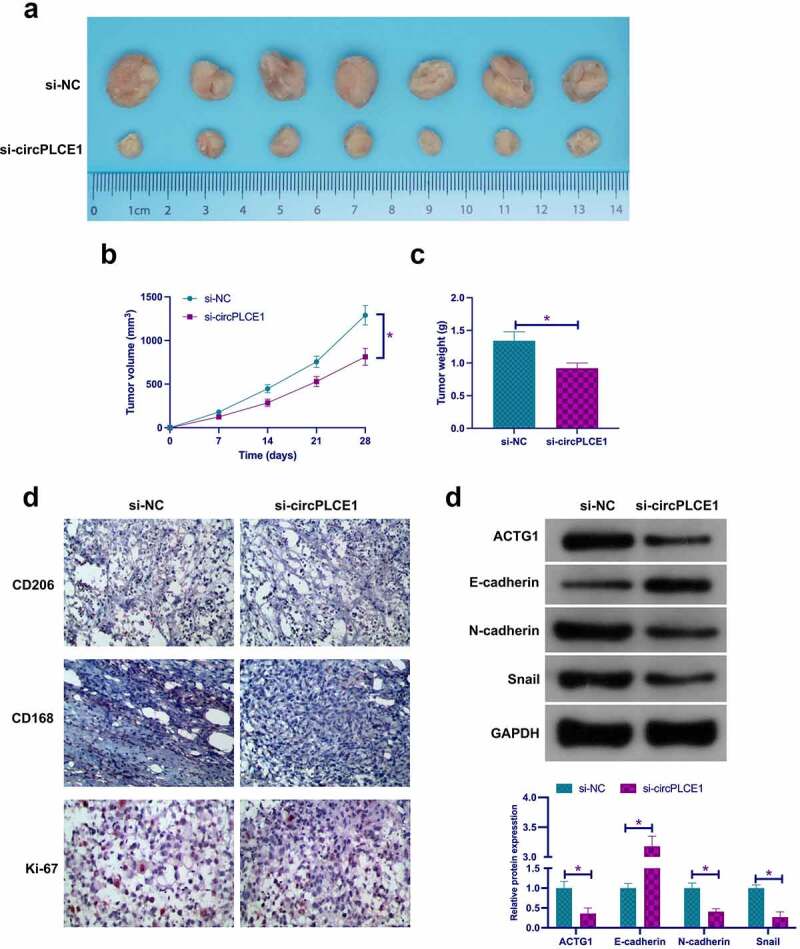
**Depressive circPLCE1 refrains tumor growth, TAM M2 polarization, and CRC EMT *in vivo*** a. Representative image of tumor; b. Tumor volume; c. Tumor weight; d. CD206, CD163 and Ki-67 detected via immunohistochemistry; e. In tumor ACTG1, E-cadherin, N-cadherin and Snail detected via Western blot. Expression of the data was as mean ± SD (n = 7); *P < 0.05.

To support the *in vitro* results, performance of *in vivo* experiments was for validation. As clarified in Figure 8(a-e), circPLCE1 depression reduced tumor growth including suppressive tumor volume, the proportion of Ki-67, CD206 and CD163 positive cells, ACTG1, N-cadherin and Snail, but motivated E-cadherin. Shortly, Refrained circPLCE1 curbed tumor growth *in vivo*, TAM M2 polarization, and CRC EMT.

## 4 Discussion

Nowadays, the unpleasing long-term survival prognosis of CRC patients is principally limited via the treatment [31]. Therefore, it is crucial to further understand the mechanisms by which CRC appears and develops. Recently, a great many studies have clarified that circRNA is crucial in the development of CRC, but its specific mechanism has not been fully figured out. In this study, it was further testified the biological role of the brand-new circRNA PCLE1 in CRC macrophage polarization, glycolysis and EMT, manifesting that circPLCE1 expedited TAM M2 polarization, and CRC EMT and glycolysis via competitively binding miR-485-5p to mediate ACTG1.

Plenty of studies have clarified the potential of discrepantly expressed circRNA in the serum of cancer patients as biomarkers for cancer diagnosis and prognosis [32]. In this study, it was affirmed that circPLCE1 is elevated in CRC tissues and cells and linked with lymph node metastasis in CRC patients, which is consistent with previous results [20]. It was speculated that circPCLE1 might be a biomarker for the diagnosis and prognosis of CRC. Although the biological mechanism of circPLCE1 in CRC has been partially clarified, it is momentous to further analyze circPLCE1 in serum of CRC patients in subsequent studies. A former study has clarified that circPCLE1 expedites CRC cell proliferation but represses apoptosis, which is consistent with the results of this study [20]. This offers more sufficient data to sustain circPCLE1 performing as a proto-oncogene in CRC.

To explore whether circPCLE1 in CRC cells could impact TAM polarization in tumor microenvironment, culture of macrophages was in SW480 cell CM with repressive circPCLE1, manifesting that reduced circPCLE1 expedited TAM polarization into M2 type. Numerous studies have affirmed that long non-coding RNA (lncRNA) can control TAM polarization in CRC. For example, lncRNA HLA-F-AS1 expedites CRC metastasis by stimulating PFN1 in the exocytosis of CRC cells and mediating macrophage polarization [33]. LncRNA RPPH1 expedites CRC metastasis via interacting with TUBB3 and motivating exosome-mediated macrophage M2 polarization [34]. Nevertheless, the role of circRNA in TAM polarization remains uncertain. In this study, the first exposure of the role of circRNA was in CRC TAM polarization. Elevation of circPCLE1 expedites the polarization of TAM into M2-type and represses its polarization to M1-type, which may be conducive to the proliferation and distal metastasis of CRC. A study has clarified that lncRNA XIST expedites the proliferation and metastasis of breast and ovarian cancer via motivating the polarization of TAM into M2 type [35]. This clarifies that targeting non-coding RNAs in the tumor microenvironment rather than in cancer cells may be influential in modulating tumor growth or metastasis. Nevertheless, the expression and biological function of circPCLE1 in TAM are still ambiguous, which requires to be explored in subsequent studies.

Glucose reprogramming is an elementary feature of cancer cells. Unlike normal cells, cancer cells like metabolizing glucose through glycolysis better, thereby enhancing glucose uptake and lactate production [36]. The energy gained via glycolysis is advantageous to cancer cell proliferation, invasion, migration, EMT, and distal metastasis [37]. Former studies have clarified that circNOX4 [38], circ0136666 [39], circTADA2A [40] and other circRNAs are momentous in the glycolysis of CRC. A novel circRNA PCLE1 was supplemented in this study, and motivated glycolysis in CRC, assuring that strengthened circPCLE1 metabolizes more glucose and does not offer sufficient energy for proliferation and metastasis of CRC. A former study has reported that ACTG1 expedites glycolysis in hepatocellular carcinoma [29]. Notably, exploration of the mechanism by which circPCLE1 modulated CRC glycolysis manifested that it functioned through the competitive adsorption of miR-485-5p to mediate ACTG1, which further emphasized that ACTG1 is vital in cancer glycolysis and supposed to be a new target for repressing cancer glycolysis later.

## 5 Conclusion

In general, the role of circPCLE1 was further clarified in the biological process of CRC. It is regarded as a competitive endogenous RNA of miR-485-5p and mediates ACTG1 to expedite TAM M2 polarization, and CRC EMT and glycolysis. CircPCLE1/miR-485-5p/ACTG1 axis is supposed to be a potential molecular target for CRC treatment later.
